# Neonatal hypoglycemia in dogs—pathophysiology, risk factors, diagnosis and treatment

**DOI:** 10.3389/fvets.2024.1345933

**Published:** 2024-05-02

**Authors:** Kárita da Mata Fuchs, Keylla Helena Nobre Pacífico Pereira, Gleice Mendes Xavier, Júlia Cosenza Mendonça, Renata Oliveira Barreto, Renata Cesar Silva, Fabiana Ferreira de Souza, Maria Lucia Gomes Lourenço

**Affiliations:** ^1^Veterinary Neonatology Research Group, Department of Veterinary Clinic, School of Veterinary Medicine and Animal Science, São Paulo State University – Unesp, Botucatu, Brazil; ^2^Department of Veterinary Surgery and Animal Reproduction, School of Veterinary Medicine and Animal Science, São Paulo State University – Unesp, Botucatu, Brazil

**Keywords:** glycemia, glycemic homeostasis, metabolism, puppy, dog

## Abstract

Hypoglycemia is the most common metabolic alteration in the clinical routine of newborn dogs, acting as a predictor of mortality in these patients. The neonatal dog shows hepatic insufficiency and homeostatic mechanisms not yet fully developed, with limited glycogen reserves and limited capacity to perform glycogenolysis and gluconeogenesis. These physiological particularities make newborn dogs particularly susceptible to hypoglycemia when of fasting, even for short periods. Several maternal and neonatal factors may be related to a higher risk of developing hypoglycemia in neonates. This paper reviews glycemic homeostasis, the pathophysiology of neonatal hypoglycemia, the main causes involved and the diagnostic and therapeutic approaches to this condition.

## 1 Introduction

Currently, the canine perinatal mortality rate is around 20%, occurring mainly at the late gestational age, during and immediately after delivery, and seven days after birth (1–6). The commonly described causes are perinatal hypoxia, maternal factors, infections, malformations, hypothermia and neonatal hypoglycemia (1, 5, 7).

The neonatal period in dogs can be described as the first three to four weeks of age (3, 8, 9). During this period, the neonate presents immaturity of several organic systems, having unique characteristics that completely differ from an adult animal (5, 8).

Canine neonates are considered altricial, which means that they have some limitations at birth, increasing susceptibility to hypoglycemia (10–12). Furthermore some physiological characteristics can also contribute to the establishment of the condition. Due to hepatic immaturity, dogs are born with limited glycogen reserves and minimal gluconeogenesis capacity (8, 13, 14). In puppies that are not suckling, blood glucose can drop rapidly, as the ability to maintain normoglycemia in cases of fasting is reduced. Hepatic reserves will be completely depleted within 24 h; however, a rapid decline in blood glucose may occur before this period in frail, sick, premature, or low birth weight neonates (5, 7, 13).

Hypoglycemia in newborns can have serious consequences, such as encephalopathies, since the body-to-brain ratio in newborns is high, in addition to the high metabolic rate, requiring a greater supply of glucose (15). Glycemia is a predictor of mortality in neonatal dogs, blood glucose concentrations <92 mg/dL in the first 24 h of life increase the risk of death during the neonatal period (4). This review addresses the mechanisms of glycemic homeostasis, the pathophysiology of neonatal hypoglycemia, risk factors and presents an approach for the diagnosis, treatment and prevention of this condition.

## 2 Glucose homeostasis and the pathophysiology of neonatal hypoglycemia

Such as oxygen, glucose is essential for newborn brain function, in most conditions it is the only energy molecule consumed by the central nervous system. When it is at low levels, short, medium and long term neurological alterations can be expected. For the correct functioning of the neurological system, it is required a constant supply of glucose (15–17). The elevated neonatal energetic requirement, 20–26 kcal/100 g (1), is directly related the higher encephalic mass when compared to other stages of life (18). In canine neonates, the high body surface/ corporal mass ratio, immature thermoregulation and elevated metabolic rates also demand high amounts of energy (8). A Functional glycemic regulation system is essential.

Before birth, the fetus receives glucose via the placenta by facilitated diffusion, through glucose transport proteins (GLUT-1), however, there is no significant production of this molecule at this stage of life (17, 19). In human neonates, about 40% of the glucose taken up is converted into glycogen, which is essential for glycemic homeostasis after birth (20). Adaptation to extrauterine life involves the glycogen mobilization (glycogenolysis) and the use of alternative sources for obtaining glucose (gluconeogenesis). The series of events that ensure glucose availability during fasting is called glycemic counter-regulation (21).

Glycogenesis (conversion of glucose to glycogen) and glycogenolysis are enzymatically regulated by a cascade driven by cyclic-AMP (cAMP). Many hormones are involved in the process of glycemic regulation, such as insulin, glucagon, somatostatin, growth hormone, amylin, catecholamines (adrenaline and noradrenaline), glucocorticoids, estrogen, among others. Insulin and glucagon are the main ones, both produced in the pancreas (17, 22).

In adult animals, insulin acts to reduce glycemic indexes, allowing glucose to be removed from the bloodstream, cross the cell membrane and provide energy to all cells in the body. Glucose is readily used to synthesize ATP through a process called glycolysis. ATP is the body’s main fuel. Despite many cell types using fat as their primary energy source, red blood cells and neurons depend almost exclusively on glucose for their energy requirements, as does muscle. This hormone also acts by stimulating hepatic glycogenesis (glycogen formation) through the activation of enzymes such as glucokinase and glycogen synthase (17, 18, 23).

Glucagon secretion is directly related to blood glucose, this hormone stimulates liver cells to convert glycogen into glucose, increasing in times of hypoglycemia and regulating glycogenolysis (glycogen breakdown) and gluconeogenesis (synthesis of glucose from substrates such as lipids and proteins). Cortisol is the main hormone that acts in this regulation, stimulating glycogenolysis and hepatic gluconeogenesis, raising blood glucose (17, 23).

Therefore, the maintenance of glycemic homeostasis is based on the counter-regulatory mechanisms of hyperglycemic hormones (adrenaline and glucagon), which in cases of hypoglycemia will activate adenylate cyclase to initiate glycogenolysis, and hypoglycemic agents (insulin), which will initiate glycogenesis (20).

However, in dogs, birth is accompanied by immaturity of organs and homeostatic mechanisms essential for the life of the neonate (2, 24). The newborn dog’s liver, as well as its metabolic functions, are not fully developed at this time, hindering the biotransformation of drugs and glycemic homeostasis. The organ will only be fully developed between approximately four and five months of age (2, 8, 25). The neonate has limited capacity for glycogenolysis and gluconeogenesis. In addition, the limited reserves of hepatic glycogen, little muscle mass, scarce adipose tissue and less use of free fatty acids as an alternative source of energy, put canine newborns at great risk of developing hypoglycemia in the presence of fasting, even of short duration. Compromised gluconeogenesis by delayed maturation and induction of low-activity gluconeogenic enzymes results in hypoglycemia in human neonates, and similar mechanisms are suspected to be involved in neonatal dogs (26).

Newborn puppies from healthy, well-fed mothers are better able to maintain blood glucose levels for up to several hours after fasting. The neonate’s capacity for glycemic regulation may be directly related to the mother’s nutritional status during pregnancy (26).

In humans, newborn glycemic levels correspond to approximately 60–70% of maternal levels (15, 18, 27, 28). In newborn dogs, however, glycemic levels at birth correspond to approximately 90% of maternal levels (29). This highlights the importance of assessing maternal blood glucose at the time of delivery and replacing it, if necessary. This conduct can be beneficial for the birth of normoglycemic puppies, since often, after the cesarean section, there is a prolonged time until breastfeeding occurs, given the anesthetic recovery of the female. This fact leads to a greater chance of developing neonatal hypoglycemia (29–32). Nneonatal normoglycemia in dogs can be considered between 90 and 200 mg/dL (5).

**Figure 1:**
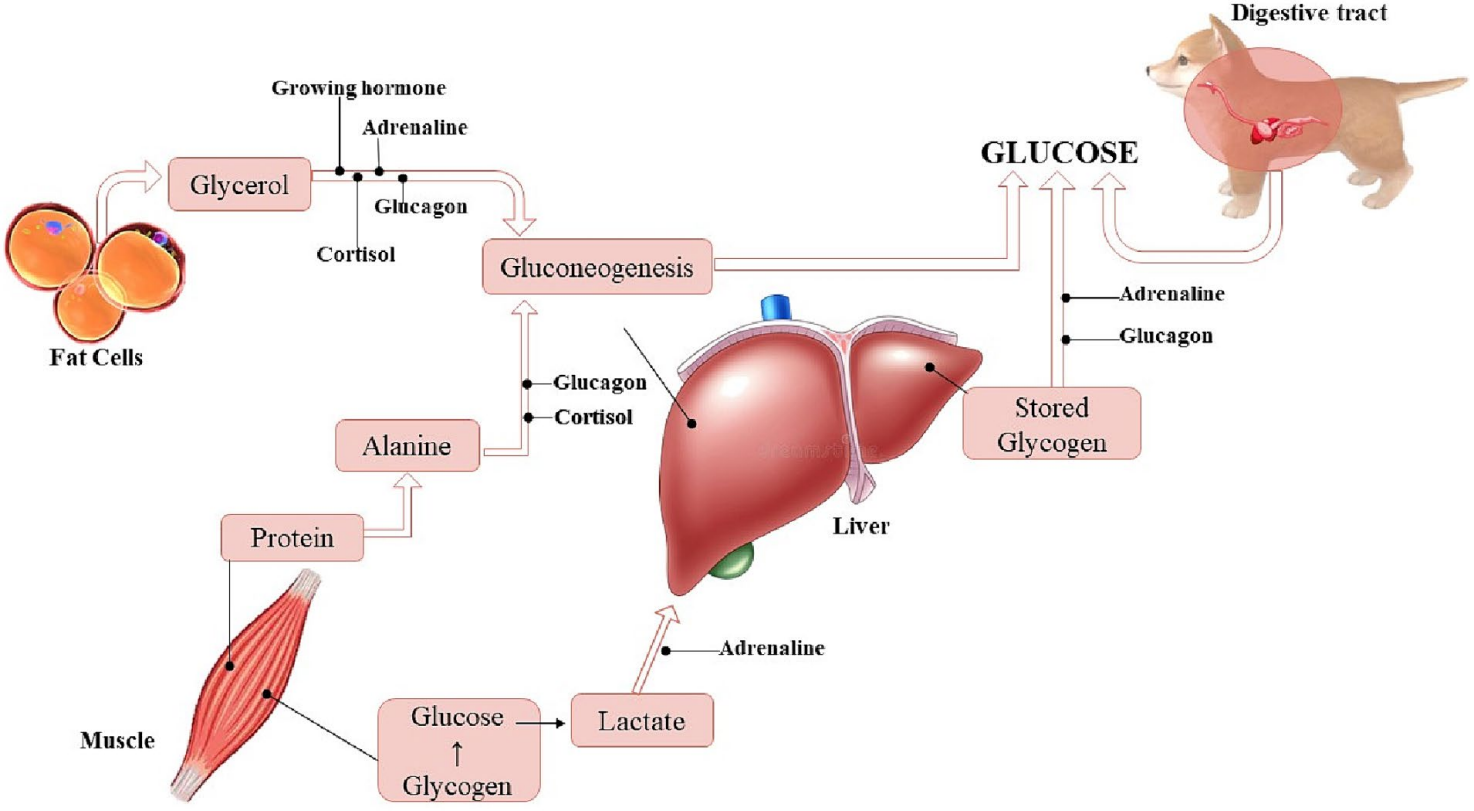
Sources for acquiring glucose in dogs (Illustration: Xavier, G. M.).

Hepatic glycogen can be observed in small amounts in the early stages of pregnancy in humans, as well as glycogenolysis enzymes (glycogen phosphorylase, transferase and debranching enzyme), increasing according to the proximity of delivery, being used by the neonate immediately after birth, rapidly decreasing its reserve. The energy from the oxidation of glucose provides about 70% of the energy needed by the patient, requiring the use of other substrates for oxidative metabolism such as amino acids, lactate, fatty acids and ketone bodies (20, 33) (Figure 1). Pups from mothers that underwent starvation for 72 h had lower liver glycogen and circulating glucose reserves than pups from the control group. In the starvation group, hepatic glycogen reserves of about 595 μmol/g were observed and in the control group, 701 μmol/g were observed (34).

In humans, it is observed that lactate works as a glucose saver and has an important role in the immediate period after birth (16). It is present in concentrations higher than normal in the first hours of life, as well as in newborn dogs (32, 35). It is assumed that this is the substrate that maintains the newborn in good health (asymptomatic) in the short term in cases of hypoglycemia (20). In studies with asymptomatic human patients, long-term neurological alteration has already been identified in about 20% of those affected, in symptomatic patients this rate reaches 35% (36), bringing the conclusion that the absence of clinical signs does not diminish the importance of the condition. Although the brain of all animals depends primarily on glucose for energy, lactate and ketones can be used as a substitute. However, the scarcity of body fat prevents ketone bodies from being a substitute energy substrate for neonates (13, 37).

Colostrum is mostly composed of lipids and carbohydrates, which are important sources of energy (19), for this reason, it is useful for containing hypoglycemia, in addition to other benefits (38). Another important source of glycogen in adults is muscle. The rate of alanine release from muscle determines the rate of gluconeogenesis during starvation. However, in newborn dogs, the reserve is minimal, due to reduced muscle mass and immature enzymatic mechanism to initiate gluconeogenesis, and thus hypoglycemia develops more easily (26). Small breeds are even more predisposed to developing hypoglycemia, as they have much smaller muscle mass. This greater predisposition can last up to three to four months of age (time for complete liver maturation), this alteration is called juvenile transient hypoglycemia and is frequently reported in toy size dogs (39).

In general, when starvation occurs in the immediate neonatal period, the main way of obtaining glucose in dogs is glycogenolysis. As the hepatic reserve of glycogen is limited in this period, it reaches less than 50% of the reserve in about three hours of life. Liver glycogenesis fails in the face of hypoglycemic scenarios in the neonate, evidencing the insufficiency of glycemic control mechanisms due to immaturity (2, 8, 40). In this phase, serum levels of insulin and glucagon and the body’s response to these hormones are unsatisfactory, making hormonal regulation inefficient, which can quickly cause hypoglycemia. Hypoglycemia is established when the serum glucose rate is reduced in relation to its tissue uptake (15, 19, 40). Although physiological immaturity poses a risk for hypoglycemia and a challenge for neonatal survival, it is important to emphasize that newborn dogs are able to maintain glycemic homeostasis under adequate nutrition (breastfeeding) and health conditions.

## 3 Risk factors for neonatal hypoglycemia in dogs

Hypoglycemia is the most common metabolic alteration in newborn patients. In veterinary medicine, the puppies most likely to manifest hypoglycemia are neonates who have gone through stressful events such as hypoxia at birth, neonates born from hypoglycemic mothers, sick, orphaned, premature and low birth weight neonates (5, 29, 41). Table 1 shows the main risk factors for the development of neonatal hypoglycemia.

**Table 1:** Main risk factors for the development of neonatal hypoglycemia (adapted from Pereira et al. (5)).

Maternal factors	Neonatal factors
Prolonged deliveries	Hypoxia at birth
Dystocias	Prematurity
Pregnant fasting/hypoglycemia	Low birth weight
Cesarean section/waiting time for maternal anesthetic recovery	Fasting
Agalactia/ hypogalactia	Weak or absent suction
Rejection/ failure of maternal instinct	Orphanhood
Malnutrition	Hypothermia
Advanced age	Infections
Diabetes mellitus	Numerous litter
	Malformations

A study has shown that about 15% of dogs can be born hypoglycemic and about 66% of dogs born by cesarean section can develop hypoglycemia in the first hour of birth, due to prolonged fasting while waiting for maternal anesthetic recovery (29). Also, about 74% of newborn dogs that are brought in for veterinary care due to some alteration or condition are diagnosed with hypoglycemia (42).

Underweight (small size <75 g, medium size <200 g, large size <400 g) (9) or premature neonates have greater hormonal and metabolic immaturity for glycemic regulation, as well as lower hepatic glycogen reserves. These newborns may also have a weak or absent sucking reflex and may not be able to ingest milk adequately. Also, low birth weight pups may not be able to compete for breastfeeding with the larger and more robust newborns in the litter, increasing the risk of hypoglycemia (5).

Sick or hypothermic neonates may show clinical depression, apathy, reduced sucking reflex and consequent reduction in milk intake, and may develop hypoglycemia. Errors in the nutritional management of orphaned pups (inadequate amount of milk offered) and maternal alterations, such as agalactia, hypogalactia, rejection or failure of the maternal instinct, also generate a feeding deficiency. Pregnancy malnutrition can lead to a reduction in neonatal hepatic glycogen stores at birth and, consequently, a greater predisposition to hypoglycemia. Congenital malformations such as cleft palate, cleft lip and macroglossia can interfere with suckling and adequate milk intake. In addition, systemic infections, such as neonatal sepsis, lead to rapid glucose consumption and increased risk of hypoglycemia (1, 4, 5, 9, 43, 44). A study demonstrated that approximately 65% of neonates in sepsis develop hypoglycemia (41).

Maternal age also shows significant influence on neonatal glycemia. A study demonstrated that puppies born from bitches under four years of age have an average glucose concentration of 125 mg/dL. In newborns of bitches between four and six years of age, mean neonatal glycemia is 97 mg/dL, while in bitches over six years of age, the average glucose concentration of the puppies is 82 mg/dL. The older the bitch, the greater the chance of neonatal hypoglycemia. Therefore, maternal age is considered a risk factor for lower neonatal survival, as glucose concentrations below 92 mg/dL in newborns are correlated with high mortality in the neonatal period (4).

In cesarean sections, some factors can lead to neonatal hypoglycemia. One of the reasons is related to the prolonged time of preoperative maternal fasting, since maternal blood glucose correlates with fetal blood glucose (27, 29–31, 43, 45, 46). Another factor occurs due to the surgical procedure and anesthetic recovery of the parturient, newborns may go through a period of prolonged food deprivation, which leads to a gradual drop in the patients’ blood glucose (27–29, 32, 47). Transient agalactia or hypogalactia may also occur after cesarean section, as well as in primiparous bitches, due to milk ejection disorders, impairing breastfeeding (5, 28).

Neonatal hypothermia may be a hypoglycemic generator, as well as the patient’s stay in cold environments. This is related to energy expenditure in an attempt to perform thermogenesis without the tremor reflex and peripheral vasoconstriction, only with the release of catecholamines and metabolization of brown fat (7, 44).

Dystocias and emergency cesarean sections offer greater stress to the newborn and the parturient, and may cause higher concentrations of glucose or even neonatal hyperglycemia (48). In a study with neonates from three types of delivery—eutocia, dystocia and emergency cesarean section—it was possible to observe higher postpartum serum cortisol and glycemic indices in neonates from dystocia and cesarean delivery, probably related to increased cortisol production, release of catecholamines (norepinephrine and epinephrine), suppression of insulin secretion and hepatic glucose mobilization (49). Also, patients who have experienced severe hypoxia may have higher glycemic rates at birth. This occurrence is associated with the excessive stress that the condition causes, generating the same hormonal cascade as the stress of childbirth, which culminates in greater mobilization of hepatic glycogen (50). However, if these neonates remain fasted, this mobilization of liver glycogen can lead to subsequent rapid hypoglycemia by depleting liver reserves.

## 4 Clinical signs and diagnosis of neonatal hypoglycemia

Hypoglycemic neonates may manifest vocalization, irritability, lethargy, decrease or absence of the suckling reflex and interruption of breastfeeding, aggravating the condition. This lack of breastfeeding can not only trigger hypoglycemia, but also hypothermia and dehydration, the so-called neonatal triad (9, 51).

Severe hypoglycemia can lead to bradycardia, cyanosis, seizures, coma and death (6, 25, 40, 41, 52). Bradycardia occurs due to reduced metabolism and glucose supply to the myocardium. As the treatment is carried out and the blood glucose increases, the heart rate increases concomitantly (41, 53). Seizures and coma are consequences of encephalopathy caused by severe hypoglycemia. This occurs because the newborn brain demands a high metabolic rate, requiring a high glycemic supply (15). Seizures in neonatal patients can be identified by muscle rigidity or pedaling movements, opisthotonos, and sialorrhea.

However, some hypoglycemic animals may be asymptomatic. A study demonstrated that hypoglycemic puppies (<90 mg/dL to 60 mg/dL) did not show relevant clinical signs (29). The observation of clinical signs such as a decrease in the Apgar score or neonatal reflexes, seizures and coma, is usually associated with very low glucose concentrations: <40 mg/dL, considered severe hypoglycemia (32). However, asymptomatic hypoglycemia should not be disregarded.

The assessment of the newborn and determination of blood glucose should be established as a priority in the initial moments after birth, especially in patients at risk (29, 54), as well as in any neonatal clinical care, supporting the determination of the appropriate conduct (54). Hypoglycemia can lead to general destabilization of the patient (25), and this condition, even if transient, can be a prognostic factor for mortality (4, 47, 54–56).

A correlation between the newborn’s weight and thermostability has already been demonstrated, as the body mass index determines the thermoregulation capacity (57, 58), in addition to the available glycogen reserve and colostrum intake. Infrared thermography has been used to assess tissue temperature indirectly, enabling the identification of hypothermia non-invasively, an important change that is associated with neonatal mortality (44, 59).

The glycemic reference value in newborn dogs is still controversial. A study described a high mortality rate in patients with blood glucose concentrations below 92 mg/dL (4), but concentrations such as 76 mg/dL (39) and 65 mg/dL (47) were also described as reference values (normoglycemia). In older studies, lower concentrations, such as 40 mg/dL, were considered normal (25). However, a recent study demonstrated a mean glycemic level of 125 mg/dL (range 106–182 mg/dL) in 349 healthy neonatal dogs during the first four weeks of age (41). As a glucose concentration < 92 mg/dL during the first 24 h of life is correlated with a higher risk of death during the first 21 days of age (4), it is understood that concentrations lower than this are no longer considered normal. Thus, neonatal normoglycemia in dogs can be considered between 90 and 200 mg/dL (5).

**Figure 2:**
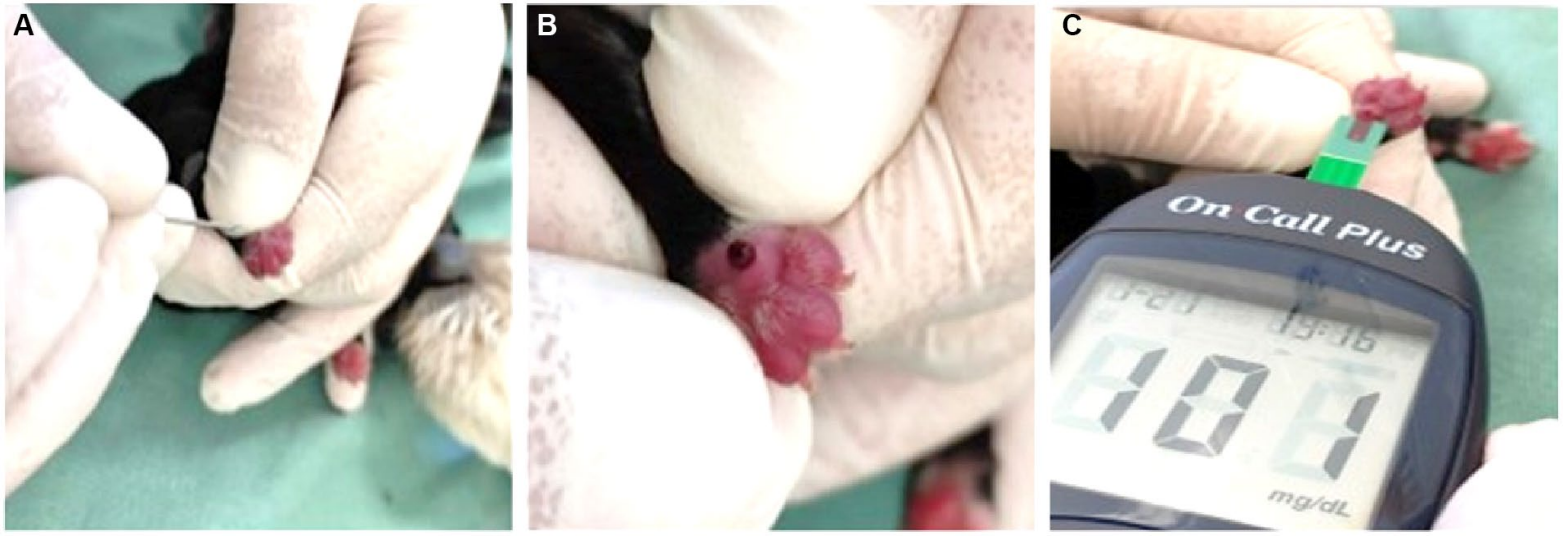
**(A)** Puncture of the paw pad in a neonate with a 30 × 0.7 mm needle. **(B)** Formation of the drop of blood on the cushion. **(C)** Assessment of blood glucose using a glucometer.

Glycemia can be assessed with a portable glucometer, using a 30×0.7 mm hypodermic needle to collect a drop of blood from the newborn’s metacarpal and/or metatarsal pad (Figure 2) or from the inner portion of the ear, or even collecting blood from the jugular vein. Based on the clinical signs presented, neonatal hypoglycemia can be classified into as mild (<90 to 70 mg/dL), moderate (<70 mg/dL to 40 mg/dL), and severe (<40 mg/dL) (4, 29, 32).

## 5 Treatment

**Figure 3:**
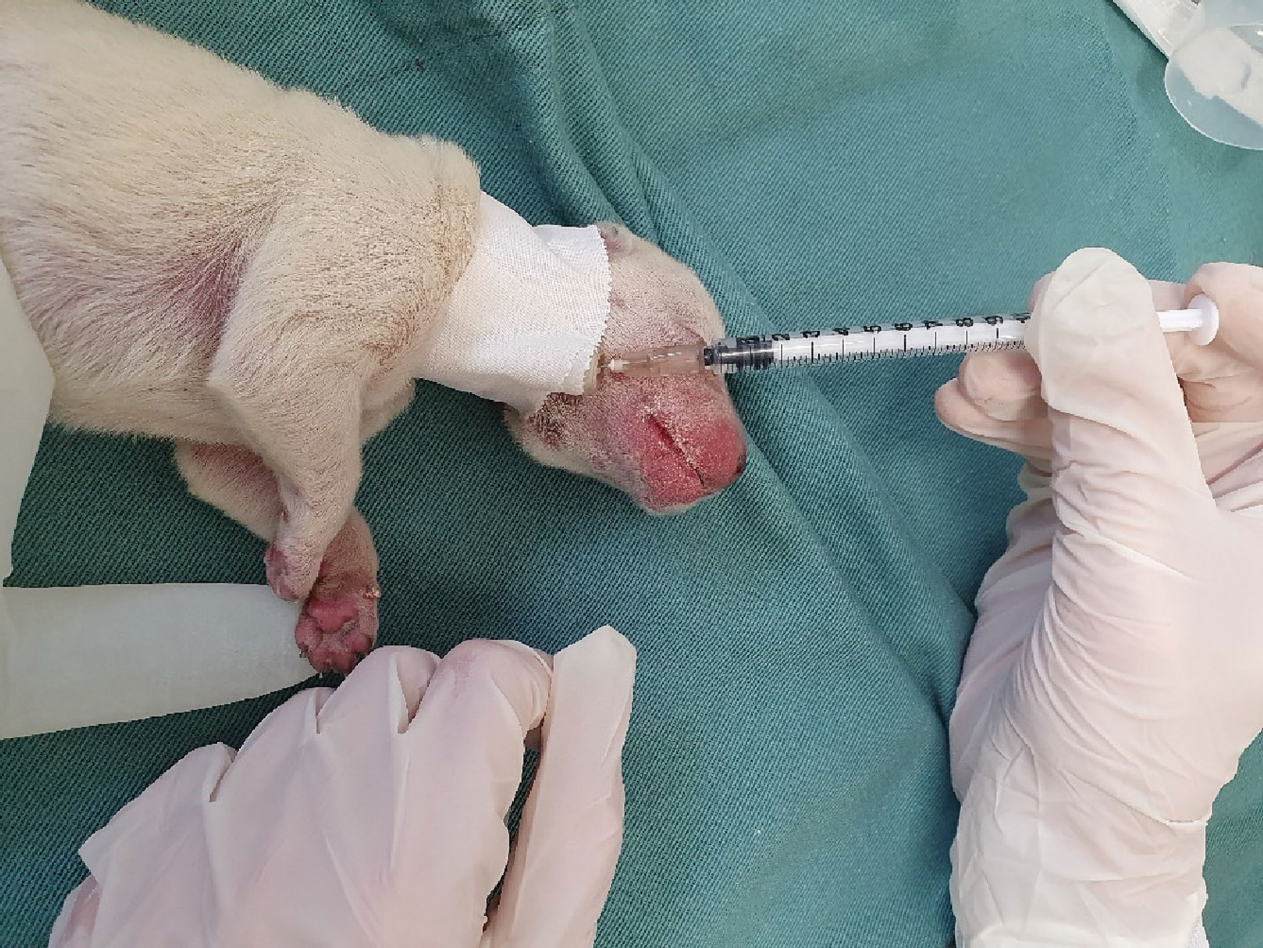
Administration of intravenous glucose through the jugular vein in a newborn dog. In the image, the jugular vein was cannulated with a 24-gauge catheter, connected to an adapter plug (PRN) and secured with adhesive tape around the patient’s neck.

With confirmation of hypoglycemia, treatment should be carried out immediately. Glycemic replacement can be performed with 10% or 12.5% glucose (the latter obtained by diluting 50% glucose in sterile water in 1:3 ratio), volume of 0.2 to 0.5 mL per 100 grams of weight, intravenously (Figure 3) or intraosseously, slowly, to avoid rebound hypoglycemia. Continuous infusion with 5% glucose should be performed in patients who are unable to maintain a stable glycemia after initial glycemic replacement. Maintenance can be performed at a volume of 6 to 18 mL per 100 grams of weight in 24 h (9, 13, 41).

The administration of oral glucose is the slowest form of glycemic replacement; therefore, it should be avoided in patients with severe hypoglycemia, being recommended the intravenous and intraosseous routes. Oral glycemic replacement, in puppies with mild to moderate hypoglycemia, can be performed using bottles or syringes, and in case of weak or absent neonatal sucking, glucose should be administered through an orogastric tube (a urethral catheter No. 4 or 6 is commonly used for this function in newborn dogs). The tube must be measured from the tip of the nose to the last rib and marked with a tape at this limit. The mark will guide the tube insertion length correctly into the stomach, preventing the tube from kinking into the gastrointestinal tract. The procedure is performed by placing the newborn in ventral decubitus and holding his head, the probe must be inserted in the mouth, observing if there is no resistance in the path. Generally, the neonate will swallow the tube easily. If the tube is accidentally inserted into the trachea, it will not be inserted until the marking has been made, as it will only reach the bifurcation of the trachea (causing resistance along the way). Therefore, if resistance is felt when inserting the tube into the mouth, it should be removed and the procedure should be tried again. Oral supplementation should only be performed in normothermic neonates (9, 13, 56).

A study demonstrated that the use of hypercaloric supplements with vitamins, carbohydrates, fats and amino acids can be used for oral neonatal glycemic replacement, replacing glucose, and bringing nutritional benefits to the puppy. In its composition, the hypercaloric supplement must provide an average of 43% carbohydrates and 35% fat (29).

The empirical administration of oral glucose in newborn dogs at birth is often observed, however, this conduct is contraindicated, since it can induce neonatal hyperglycemia (41, 44, 52). Glucose should only be offered to newborns after glycemic evaluation and diagnosis of hypoglycemia.

## 6 How to prevent neonatal hypoglycemia?

It is important to provide adequate nutrition to the parturient to prevent neonatal hypoglycemia. The formation of fetal liver glycogen stores and the capacity for neonatal glycemic regulation in the postpartum period will depend on the mother’s nutritional status (26). Puppies born from bitches that underwent starvation for 72 h before giving birth had lower birth weight, lower glycemic indexes and hepatic glycogen reserves (34). It is also important to evaluate maternal glycemia in the prepartum period, the diagnosis of hypoglycemia and glycemic correction of the pregnant woman reduce the chances of puppies being born with hypoglycemia (29).

The glycemia of neonates should be accessed at birth and in the first hour of life, since the infant’s fasting while waiting for the transoperative period and the mother’s anesthetic recovery from the cesarean section is considered a risk factor for neonatal hypoglycemia (29).

It is essential to ensure adequate breastfeeding of the newborn to prevent hypoglycemia. The mother’s milk production must be constantly monitored and the presence of sucking reflex in neonates evaluated. In puppies with weak or absent suction, milk should be supplied by orogastric tube until adequate suction is restored (5).

It is important to assess the daily weight gain of neonates (using a digital scale with a scale in grams), expecting a minimum weight gain of 5 to 10% per day of initial weight. The weight must be recorded on evaluation sheets and can be easily performed by tutors or breeders. Constant weight gain is an indication of health and well-being in neonates, with weight loss being related to a breastfeeding problem or a neonatal sickness. Often, failure to gain weight is noticed before the development of clinical signs of hypoglycemia or disease, so early intervention can be performed, which is essential for a higher neonatal survival rate (5, 52).

## 7 Final considerations

Hypoglycemia is a common metabolic affection in newborn dogs. It is essential to know the physiological particularities of the newborn and the risk factors that predispose puppies to this condition, such as infectious causes, congenital malformations, low birth weight, low body temperature, failure of maternal instincts, among others.

The monitoring of neonatal and maternal glycemia at strategic moments and a quick and effective conduct in cases of hypoglycemia increase the chance of survival of the newborn.

Maternal, neonatal nutritional management and monitoring the patient after birth is essential to reduce cases of hypoglycemia and the neonatal mortality rate in dogs.

## Author contributions

KF: Conceptualization, Data curation, Formal analysis, Funding acquisition, Investigation, Methodology, Project administration, Resources, Validation, Visualization, Writing – original draft, Writing – review & editing. KP: Conceptualization, Formal analysis, Funding acquisition, Investigation, Methodology, Project administration, Resources, Validation, Visualization, Writing – original draft, Writing – review & editing. GX: Formal analysis, Visualization, Writing – review & editing. JM: Methodology, Visualization, Writing – review & editing. RB: Validation, Visualization, Writing – review & editing. RS: Methodology, Visualization, Writing – review & editing. FS: Data curation, Methodology, Supervision, Visualization, Writing – review & editing. ML: Conceptualization, Formal analysis, Funding acquisition, Investigation, Methodology, Project administration, Resources, Supervision, Validation, Visualization, Writing – review & editing.
